# Prognostic value of soluble suppression of tumorigenesis-2 (sST2) for cardiovascular events in coronary artery disease patients with and without diabetes mellitus

**DOI:** 10.1186/s12933-021-01244-3

**Published:** 2021-02-19

**Authors:** Man Li, Lei Duan, Yulun Cai, Benchuan Hao, Jianqiao Chen, Huiying Li, Hongbin Liu

**Affiliations:** 1 Department of Cardiology, The Second Medical Center, Chinese PLA General Hospital, No. 28, Fu Xing Road, Hai Dian, Beijing, China; 2 Beijing Key Laboratory of Chronic Heart Failure Precision Medicine, Beijing, China

**Keywords:** sST2, Coronary artery disease, Atherosclerosis, Biomarker

## Abstract

**Background:**

Soluble suppression of tumorigenesis-2 (sST2) is implicated in myocardial overload and has long been recognized as an inflammatory marker related to heart failure and acute coronary syndrome, but data on the prognostic value of sST2 in patients with coronary artery disease (CAD) remain limited. This study sought to investigate the prognostic value of sST2 in patients with established CAD and its predictive value in CAD patients with and without type 2 diabetes mellitus (T2DM).

**Methods:**

A total of 3641 consecutive patients were included in this prospective cohort study. The primary end point was major adverse cardiovascular events (MACEs). The secondary end point was all-cause death. The association between sST2 and outcomes was investigated using multivariable Cox regression.

**Results:**

During a median follow-up of 6.4 years, MACEs occurred in 775 patients, and 275 patients died. Multiple Cox regression models showed that a higher level of sST2 was an independent predictor of MACEs development (HR = 1.36, 95% CI 1.17–1.56, p < 0.001) and all-cause death (HR = 2.01, 95% CI 1.56–2.59, p < 0.001). The addition of sST2 to established risk factors significantly improved risk prediction of the composite outcome of MACEs and all-cause death (C-index, net reclassification index, and integrated discrimination improvement, all p < 0.05). In subgroup analysis depending on diabetes status, the diabetes group had a significantly higher level of sST2, which remained a significant predictor of MACEs and all-cause death in patients with and without T2DM in multivariable models. The area under the curve (AUC) of CAD patients with diabetes mellitus was significantly higher than that of those without T2DM. For MACEs, the AUC was 0.737 (patients with T2DM) vs 0.620 (patients without T2DM). For all-cause death, the AUC was 0.923 (patients with T2DM) vs 0.789 (patients without T2DM).

**Conclusions:**

A higher level of sST2 is significantly associated with long-term MACEs and all-cause death in CAD patients with and without T2DM. sST2 has strong predictive value for cardiovascular adverse events in CAD patients with T2DM, and these results provide new evidence for the role of sST2.

## Background

Coronary artery disease (CAD) remains the leading cause of death worldwide [1]. Patients with previous coronary heart disease have a high probability of major adverse cardiac events (MACEs). The development of reliable prognostic biomarkers is of vital importance to patients with established CAD.

Suppression of tumorigenesis-2 (ST2) is an interleukin-1 (IL-1) receptor family member that exists in two isoforms: membrane-bound (ST2L) and soluble isoform soluble suppression of tumorigenesis-2 (sST2) forms [2]. Previous studies have suggested that IL-33 acts as an “alarm” to signal potential tissue stress or damage [3, 4]. IL-33 promotes the production of inflammatory cytokines and Th2 immune responses by signaling through a heterodimer receptor complex composed of ST2L and IL-1 receptor attachment proteins; sST2 is known to bind to IL-33, and it acts as a “decoy” receptor for IL-33 to inhibit IL-33/ST2L signaling [5, 6]. An increase in the circulating sST2 concentration attenuates the systemic biological effects of IL-33. Therefore, sST2 has long been recognized as a marker of the activation of both inflammatory and hemodynamic overload [7–9]. Subsequently, soluble sST2 has been shown to be a powerful independent prognosticator in patients with acute coronary syndrome (ACS) [10, 11], as well as heart failure (HF) [12–14]. However, it remains unclear whether sST2 is predictive of MACEs and all-cause death in long-term follow-up of CAD [13, 15–18]. In addition, type 2 diabetes mellitus (T2DM) is a known predictor of elevated sST2 [19, 20]. Thus, we performed a large-scale, prospective study to evaluate the prognostic value of sST2 for MACEs and all-cause mortality in patients with established CAD with and without T2DM over long-term follow-up.

## Materials and methods

### The study population

**Fig. 1:**
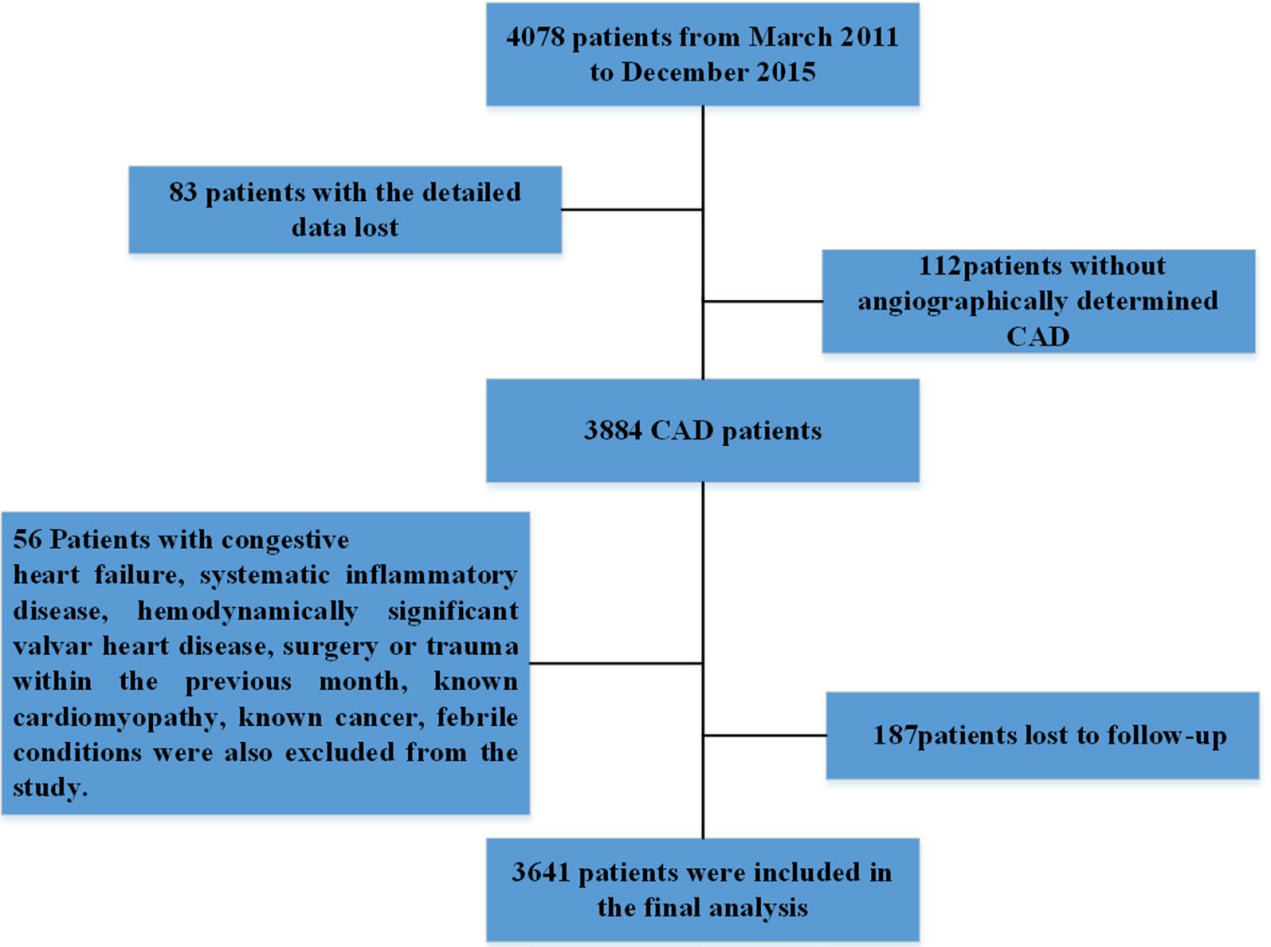
Flowchart of the study

The design, details, and primary results of this study have been previously reported [21]. Briefly, the purpose of this study was to evaluate the prognostic value of different biomarkers for adverse cardiac events in patients with CAD. From March 2011 to December 2015, 4078 patients who underwent coronary angiography at the Chinese PLA General Hospital were recruited for this study. These patients underwent coronary angiography examination because of angina-like chest pain or positive noninvasive tests (such as the treadmill exercise test or coronary computed tomography angiography). Based on the angiography results, patients with stenosis ≥ 50% in at least one major coronary artery were diagnosed with CAD. Patients were excluded if they had severe heart failure, atrial fibrillation, aortic dissection, active infectious disease, history of malignancy, or end-stage renal disease or if they were in a deep coma. In the current study, patients were also excluded if their blood samples or detailed data were not available. Finally, 3641 patients were included in the present study (Fig. 1).

### Data collection

Baseline data on demographic characteristics, lifestyle risk factors, cardiac history, and medications were collected at the time of enrollment. Blood samples were collected in the early morning. According to the protocol, blood was collected into EDTA-anticoagulated plastic tubes. All of the blood samples were centrifuged at 1000×*g* for 10 min, and plasma samples were stored at − 80 °C. Lipid profiles were measured using an automatic biochemistry analyzer (Hitachi 7150, Tokyo, Japan). Specifically, total cholesterol (TC) and triglyceride (TG) levels were analyzed by enzymatic methods. The low-density lipoprotein cholesterol (LDL-C) concentration was determined by the selective solubilization method (low-density lipid cholesterol test kit; Kyowa Medex, Tokyo, Japan). The high-density lipoprotein cholesterol (HDL-C) concentration was determined by a homogeneous method (Determiner L HDL; Kyowa Medex, Tokyo, Japan). Patients whose blood pressure was > 140/90 mm Hg and those who were treated with antihypertensive medications were considered to have hypertension. Hyperlipidemia was defined when one of the following criteria was met: LDL cholesterol ≥ 160 mg/dL; total cholesterol ≥ 240 mg/dL; triglyceride ≥ 220 mg/dL; HDL cholesterol ≤ 35 mg/dL; or use of statin medication. Diabetes mellitus (DM) was defined as the presence of diabetes symptoms and a resting plasma glucose concentration ≥ 200 mg/dL, a fasting glucose plasma concentration ≥ 126 mg/dL, or a 2-h plasma glucose concentration ≥ 200 mg/dL after a 75-g oral glucose tolerance test; or the use of a hypoglycemic agent or other medications for DM. Patients were considered current smokers if they reported any tobacco use in the previous 30 days.

### Plasma sST2 detection

Blood samples were collected within 24 h of hospital admission after at least 8 h of fasting. The sST2 levels in the plasma were determined in single measurements using a quantitative sandwich monoclonal enzyme-linked immunosorbent assay (Presage sST2 Assay, Critical Diagnostics, Inc., San Diego, California, USA). A standard curve was constructed. Analysts were blinded to the patients’ characteristics and endpoints of the study participants.

### Outcome assessment

Patients were followed-up until May 2020 or until the occurrence of cardiovascular events. All of the participants were followed up by analyses of clinical materials and telephone contact semiannually. The primary endpoint was the development of MACEs, and the second endpoint was all-cause death. A MACE was defined as cardiac death, myocardial infarction, unstable angina or unplanned revascularization. All deaths were considered to be cardiac related unless a definitive non-cardiac cause was established. Unstable angina pectoris was defined as new or accelerating symptoms of myocardial ischemia accompanied by new ischemic ST-T changes. Myocardial infarction was defined as an increase in cardiac biomarkers with evidence of myocardial ischemia. Unplanned revascularization was diagnosed if the patient underwent percutaneous coronary intervention (PCI) or coronary artery bypass grafting (CABG) with evidence of myocardial ischemia. We obtained follow-up data for all of the patients until the primary outcome or date of censoring. All-cause death was defined as death from any cause. The follow-up time was calculated from the date of cardiac event onset to the date of event occurrence or the date of the last follow-up. Written informed content was obtained from all of the study participants, and the study was approved by the ethics committee of the Chinese PLA General Hospital.

### Statistical analysis

Differences in baseline characteristics between the two groups were evaluated by the chi-square test (categorical variables) and analysis of variance, as appropriate. Variables with a normal distribution are presented as the mean ± standard deviation (SD), whereas in the case of non-normality, the medians are presented. Categorical data are presented as counts or percentages. Kaplan–Meier curves were used to estimate the cumulative incidence risks of outcomes across baseline sST2 levels, which were compared by log-rank tests. Cox proportional hazards models were used to evaluate the association of baseline sST2 levels with the study endpoints. The results are presented as the hazard ratios (HRs) and 95% confidence intervals (CIs) according to the sST2 levels. We fitted two multivariate proportional hazards models. Model 1 was adjusted for clinical variables, including age, sex, BMI, current smoking status, hypertension, hyperlipidemia, DM, previous myocardial infarction (MI), and previous PCI/CABG, TC, TG, HDL-C, and LDL-C. Model 2 was based on Model 1, with the addition of sST2. The relationship between the sST2 levels and outcomes is presented with Cox proportional hazard models both with sST2 as a continuous variable and with sST2 as a categorical variable. The area under the receiver operating characteristic curve (AUC) was used to compare the predictive ability of the parameters of interest. Furthermore, C-index, continuous net reclassification index (NRI) and integrated discrimination improvement (IDI) were calculated to evaluate any improvement in prognostic prediction when sST2 was added to the established model. SPSS software, (version 22.0) and R software, version 4.0.0 (R Foundation for Statistical Computing) were used for descriptive data analysis. All of the statistical tests were 2 tailed, and p values < 0.05 were considered statistically significant.

## Results

### Baseline characteristics

Baseline measurements of sST2 were available for 3641 patients. The median concentration of sST2 was 19 ng/mL. The baseline characteristics of the consecutive CAD patients are shown in Table 1. We divided the patients into two groups based on the median concentrations of sST2. The patients with higher concentrations of sST2 were older and more often men, had a higher prevalence of previous PCI/CABG, and were more likely to have ACS. These patients also had higher TG levels (Table 1).

**Table 1:** Baseline clinical and laboratory characteristics of the study patients according to sST2 levels

	Total n = 3641	sST2 (< 19 ng/mL) (n = 1818)	sST2 (≥ 19 ng/mL) (n = 1823) (n = 1823)	p value for trend
Age, years	61.40 (26–95)	61.03 (26–93)	61.86 (30–95)	0.031
Male, n%	2632 (72.29)	1226 (67.44)	1406 (77.13)	0.000
BMI (kg/m^2^)	25.64 (13.30–42.19)	25.70 (13.30–42.19)	25.60 (14.50–39.70)	0.230
Risk factors for atherosclerosis				
Current smokers, n (%)	1668 (45.81)	810 (44.55)	858 (47.07)	0.086
Hypertension, n (%)	2370 (65.09)	1160 (63.80)	1210 (66.37)	0.090
Hyperlipidemia, n (%)	1120 (30.76)	581 (31.96)	539 (29.57)	0.099
Diabetes mellitus, n (%)	1163 (31.94)	590 (32.45)	573 (31.43)	0.943
Cardiac history				
Previous MI, n (%)	254 (6.98)	125 (6.88)	129 (7.08)	0.085
Previous PCI/CABG, n (%)	299 (8.21)	127 (6.99)	172 (9.43)	0.003
Laboratory data				
TC (mmol/L)	4.03 ± 1.08	4.03 ± 1.07	4.03 ± 1.09	0.816
HDL-C (mmol/L)	1.07 ± 0.68	1.07 ± 0.71	1.07 ± 0.65	0.951
LDL-C (mmol/L)	2.40 ± 0.91	2.38 ± 0.85	2.41 ± 0.91	0.314
TG (mmol/L)	1.62 ± 1.21	1.54 ± 1.40	1.70 ± 0.98	0.000
Medications				
Aspirin, n (%)	3415 (93.79)	1712 (94.17)	1703 (93.42)	0.138
ACEI, n (%)	1503 (41.28)	720 (39.60)	783 (42.95)	0.530
β-blocker, n(%)	1821 (50.01)	891 (49.01)	930 (51.01)	0.960
Statins, n (%)	3442 (94.53)	1725 (94.88)	1717 (94.19)	0.153
CAD classification				
SAP	899 (24.69)	486 (26.73)	413 (22.65)	0.950
ACS	2742 (75.3)	1128 (62.05)	1614 (88.54)	0.030

The baseline characteristics of the study participants according to their T2DM status are summarized in Table 2. In all, 1163 (31.94%) CAD patients had T2DM. After adjusting for age and sex, the diabetes group still had a significantly higher sST2 level, a higher rate of hypertension, a higher TG level, higher BMI, a higher rate of angiotensin-converting enzyme inhibitor (ACEI) and β-blocker medication use, and a higher rate of ACS than the group of patients without diabetes. Diabetes patients also had a lower rate of smoking and lower TC and LDL-C levels.

**Table 2:** The baseline characteristics of the groups with and without diabetes

	Diabetic (n = 1163)	Non-diabetic (n = 2478)	p value for trend
Age, years	62.10 (27–92)	61.10 (28–95)	0.019
Male, n%	809 (69.56)	1823 (73.57)	0.017
BMI(kg/m^2^)	26.06 ± 3.40	25.45 ± 3.56	0.000
Risk factors for atherosclerosis			
Current smokers, n (%)	494 (42.48)	1174 (47.38)	0.007
Hypertension, n (%)	839 (72.14)	1531 (61.78)	0.000
Hyperlipidemia, n (%)	342 (29.41)	778 (31.40)	0.234
Cardiac history			
Previous MI, n (%)	81 (6.94)	173 (6.98)	0.288
Previous PCI/CABG, n (%)	95 (8.17)	204 (8.23)	0.312
Laboratory data			
TC (mmol/L)	3.96 ± 1.06	4.07 ± 1.10	0.004
HDL-C (mmol/L)	1.05 ± 0.87	1.09 ± 0.58	0.193
LDL-C (mmol/L)	2.32 ± 0.85	2.44 ± 0.94	0.000
TG (mmol/L)	1.75 ± 1.32	1.57 ± 1.15	0.000
sST2	19.98 ± 13.97	18.43 ± 13.73	0.000
Aspirin, n (%)	1085 (93.29)	2330 (94.03)	0.591
ACEI, n (%)	537 (46.17)	966 (38.98)	0.000
β-blocker, n (%)	632 (54.34)	1189 (47.98)	0.039
Statins, n (%)	1097 (94.33)	2345 (94.63)	0.918
CAD classification			
SAP	285 (24.51)	614 (24.78)	0.065
ACS	954 (82.03)	1788 (72.15)	0.000

### Association between plasma sST2 and the prognosis of MACEs and all-cause death

#### Primary endpoint

**Fig. 2:**
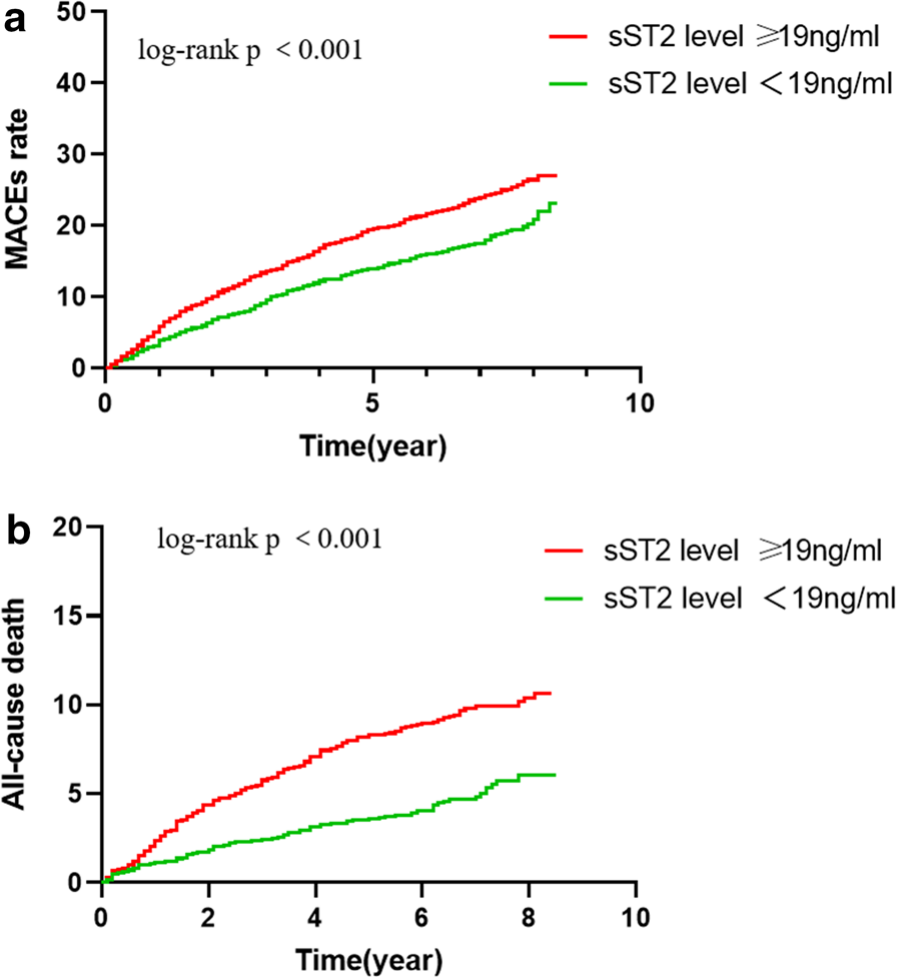
Kaplan–Meier curves for prediction of MACEs (**a**) and all-cause death (**b**) in patients with higher levels of sST2 (ST2 ≥ 19 ng/ml) and lower levels of sST2 (sST2 < 19 ng/ml)

During the median follow-up of 6.4 years, MACEs occurred in 775 (21.29%) patients. Patients with higher sST2 levels had a significantly higher rate of MACEs than patients with lower levels (24.90% vs 17.66%, p < 0.001). After adjusting for the established factors included in Model 1 and using the lower level of sST2 as a reference, we found that patients with an sST2 level ≥ 19 ng/mL had a significantly higher risk of experiencing a primary outcome (HR = 1.36, 95% CI 1.17–1.56, p < 0.001) (Table 3). Kaplan–Meier curves showed the cumulative event curves for MACEs stratified according to sST2 levels. The results showed that patients with higher levels of sST2 were more likely to have a higher MACEs rate (log-rank test, p < 0.001) (Fig. 2a).

**Table 3:** HRs and 95% CIs of outcomes according to the categories of plasma sST2 in Model 2

Outcomes	sST2 level		
	< 19 ng/ml	≥ 19 ng/ml	P trend
Primary outcome: MACEs	775		
Number of cases (%)	321 (17.66)	454 (24.90)	< 0.001
Model 2 HR	1.00 (REF)	1.36 (1.17–1.56)	< 0.001
Cardiac death	163		
Number of cases (%)	48 (2.64)	115 (6.31)	< 0.001
Model 2 HR	1.00 (REF)	1.70 (1.50–1.98)	0.033
MI	57		
Number of cases (%)	21 (1.1)	36 (1.8)	0.066
Model 2 HR	REF	1.17 (0.67–2.04)	0.579
Unstable angina	550		
Number of cases (%)	245 (13.48)	305 (16.73)	0.280
Model 2 HR	REF	1.42 (1.25–1.98)	< 0.001
Revascularization treatment	5		
Number of cases (%)	2 (0.11)	3 (0.16)	0.343
Model 2 HR	REF	1.08 (0.94–1.17)	0.265
Secondary outcome: all-cause death	275		
Number of cases (%)	90 (4.95)	185 (10.15)	< 0.001
Model 2 HR	1.00 (REF)	2.01 (1.56–2.59)	< 0.001

#### Secondary endpoint

During the follow-up, 275 (7.55%) patients died. Compared with participants with a lower level of sST2, the group with a higher level had a significantly higher incidence of all-cause death (10.15% vs 4.95%). After adjusting for the established factors included in Model 1 and using the lower level of sST2 as a reference, we found that patients with an sST2 level ≥ 19 ng/mL had a higher risk of all-cause death (HR = 2.01, 95% CI 1.56–2.59, p < 0.001) (Table 3). Kaplan–Meier curves illustrated the cumulative event curves for all-cause death stratified according to sST2 levels, and patients with higher levels of sST2 were more likely to experience all-cause death (log-rank test, p < 0.001) (Fig. 2b).

### Incremental value of sST2 over conventional risk factors

**Fig. 3:**
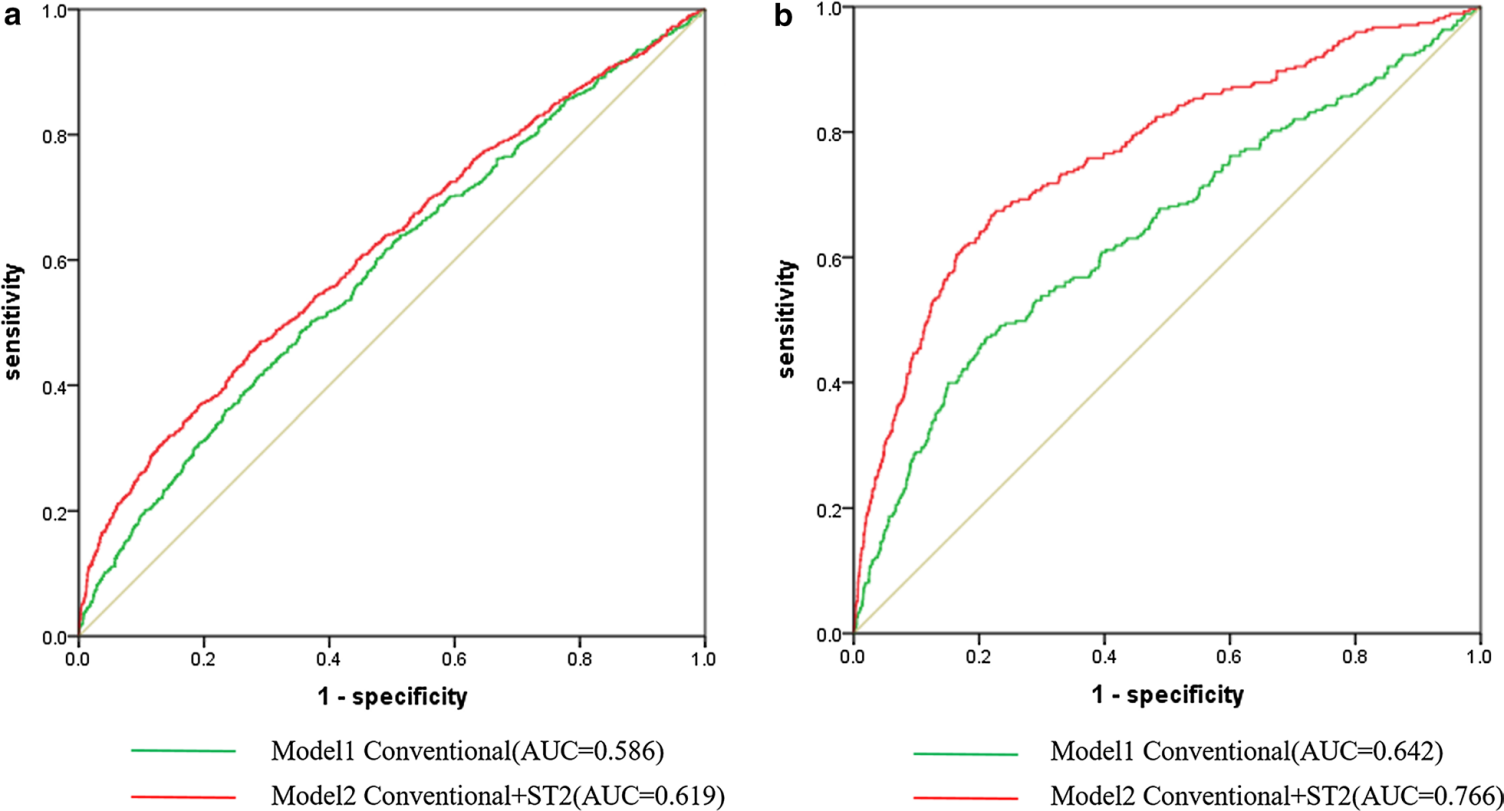
ROC curve analyses that relate sST2 levels to MACEs (**a**) and all-cause death (**b**)

For MACEs, we further examined whether the addition of sST2 to the clinical model consisting of traditional risk factors could improve the prediction performance of the risk model. As shown in Table 4, the addition of sST2 significantly improved the C-index from 0.586 (95% CI 0.559–0.603) to 0.619 (95% CI 0.605–0.638). A significant difference compared with the clinical model with sST2 (p < 0.001) was observed (Fig. 3a). Furthermore, the addition of sST2 categories to Model 1 significantly improved the NRI = 0.178 (95% CI 0.094–0.262, p < 0.001) and IDI = 0.009 (95% CI 0.003–0.014, p < 0.001) (Table 4). For all-cause mortality, adding sST2 significantly improved the C-index from 0.642 (95% CI 0.594–0.701) to 0.766 (95% CI 0.717–0.806). A significant difference compared with the clinical model with sST2 (p < 0.001) was observed (Fig. 3b). Moreover, adding sST2 categories to Model 1 significantly improved the NRI = 0.342 (95% CI 0.118–0.547 p < 0.001) and IDI = 0.012 (95% CI 0.004–0.013, p < 0.001) (Table 4).

**Table 4:** Reclassification and discrimination statistics for clinical outcomes by plasma sST2

Clinical outcomes	Model	C-index		Continuous NRI,%		IDI,%	
		Estimate (95%CI)	P value	Estimate (95%CI)	P value	Estimate (95%CI)	P value
MACEs	Model1	0.586(0.559–0.603)	< 0.001	REF	< 0.001	REF	< 0.002
	Model1 + sST2	0.619(0.605–0.638)		17.8 (9.4–26.2)		0.9 (0.3–1.4)	
Cardiac death	Model1	0.746(0.703–0.789)	< 0.001	REF	< 0.001	REF	< 0.001
	Model1 + sST2	0.783(0.743–0.823)		27.9 (15.9–34.5)		0.8 (0.2–1.6)	
MI	Model1	0.644(0.564–0.715)	0.352	REF	0.561	REF	0.721
	Model1 + sST2	0.655(0.582–0.729)		15.3 (9.7–20.0)		1.2 (0.8–2.8)	
Unstable angina	Model1	0.583(0.561–0.608)	< 0.001	REF	0.015	REF	0.001
	Model1 + sST2	0.601(0.583–0.617)		18.2 (10.4–31.4)		0.07 (0.01–0.26)	
Revascularization	Model1	0.575(0.545–0.596)	< 0.001	REF	< 0.001	REF	< 0.001
	Model1 + sST2	0.584(0.572–0.609)		16.7 (3.9–22.3)		1.4 (0.6–1.8)	
All-cause death	Model1	0.642(0.594–0.701)	< 0.001	REF	< 0.001	REF	< 0.001
	Model1 + sST2	0.766(0.717–0.806)		34.2 (11.8–54.7)		1.2 (0.4–1.3)

### The prognostic value of sST2 in CAD patients with and without diabetes

**Fig. 4:**
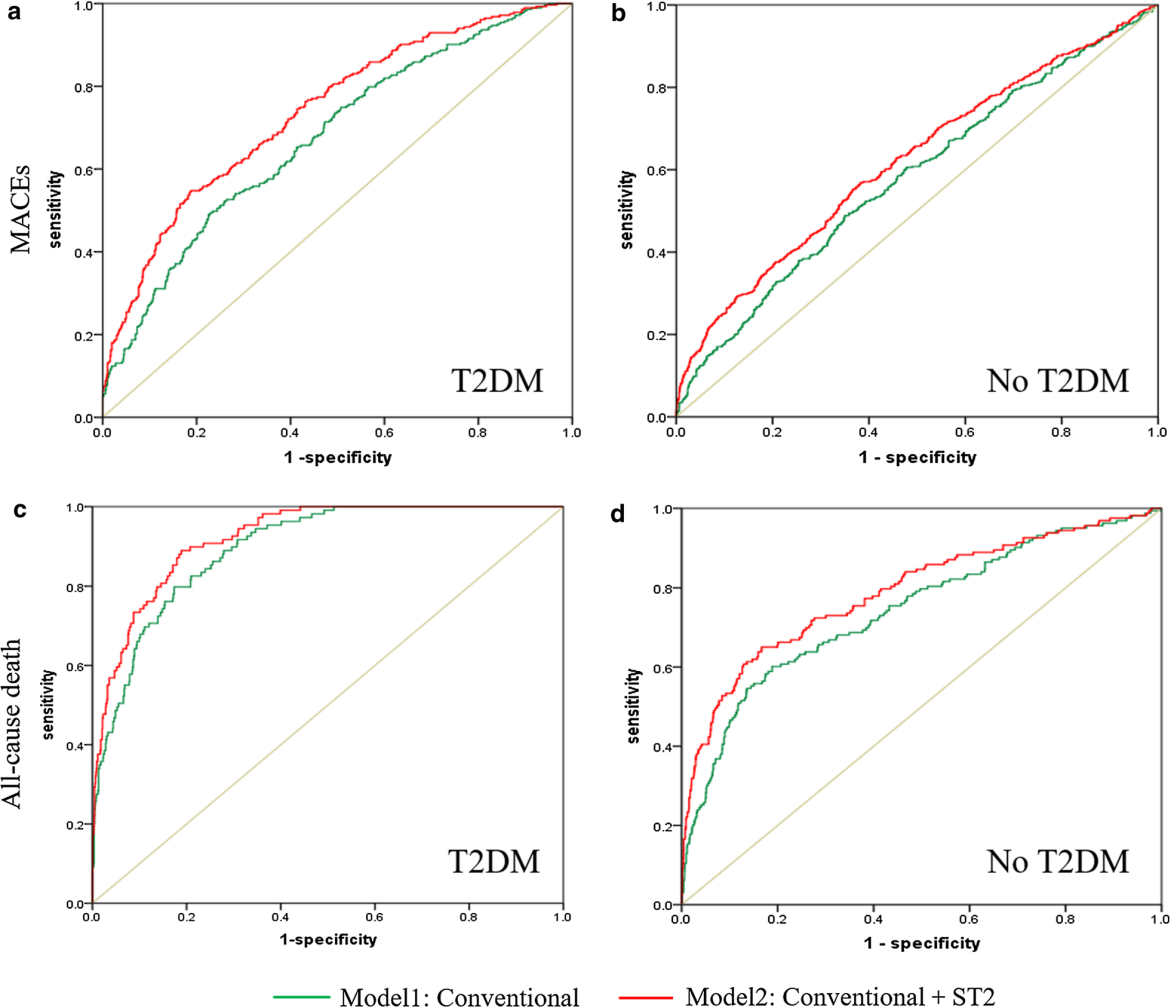
ROC curve analyses that relate sST2 levels to MACEs and all-cause death in patients with diabetes (**a**, **c**) and without diabetes (**b**, **d**)

The predictive value of sST2 for MACEs and all-cause death in CAD patients with and without diabetes is presented in Fig. 4. According to multivariable Cox regression analyses, sST2 remained a significant predictor of MACEs in patients with and without T2DM after adjusting for age, sex and other confounders. Among patients with T2DM, the AUC increased from 0.675 (95% CI 0.639–0.711) to 0.737 (95% CI 0.704–0.771) (p < 0.001). In patients without T2DM, the AUC increased from 0.581 (95% CI 0.552–0.610) to 0.620 (95% CI 0.591–0.649) (p < 0.001) (Fig. 4a, b). For all-cause death, in the multivariable Cox regression analyses, sST2 remained a significant predictor of all-cause death in patients with and without T2DM after adjusting for age, sex and other confounders. Among patients with T2DM, the AUC increased from 0.896 (95% CI 0.870–0.922) to 0.923 (95% CI 0.890–0.960) (p < 0.001). In patients without T2DM, the AUC increased from 0.744 (95% CI 0.700–0.787) to 0.789 (95% CI 0.748–0.831) (p < 0.001) (Fig. 4c, d).

## Discussion

Our study established that a higher level of sST2 was a significant and independent predictor of cardiovascular events. We found that higher concentrations of sST2 (≥ 19 ng/mL) were associated with an increased risk of MACEs and all-cause death in patients with CAD. Higher concentrations of sST2 remained an independent indicator of MACEs and all-cause mortality after adjusting for established traditional risk factors for cardiovascular disease. Furthermore, our study confirmed the incremental prognostic value of sST2 for MACEs and all-cause mortality beyond the clinical model. In subgroup analysis depending on the diabetes status, sST2 remained a significant predictor of MACEs and all-cause death in patients with and without T2DM after adjusting for age, sex and other confounders. In summary, our results suggested that the addition of plasma sST2 measurements to established cardiovascular risk factors may further improve risk stratification in patients with CAD. Our results also provided updated information about the long-term prognostic role of sST2 in patients with established CAD with and without T2DM.

### Prognostic value of biomarkers in CAD patients

Over the past two decades, biomarkers have become increasingly important tools that can help to improve patient prognosis [22–24]. Numerous biomarkers have been identified for the diagnostic, prognostic and risk prediction of cardiovascular disease, but few have been adopted in clinical practice [25]. The most extensively used cardiovascular biomarkers are natriuretic peptides for the diagnosis and prognosis of heart failure and cardiac troponins for the diagnosis of acute myocardial infarction. More in-depth experimental studies of the pathophysiology of atherosclerosis have identified a large number of molecules as potential prognostic biomarkers in cardiovascular disease [26]. To date, however, no marker has been shown to predict cardiovascular events with high accuracy. Therefore, the investigation of potential markers that can predict cardiovascular events is still of great value. Only 2 small studies have reported the prognostic value of sST2 in patients with CAD [16, 27]. One study showed that sST2 and IL-33 were associated with mortality in patients with ST elevation myocardial infarction (STEMI) but not in patients with non-STEMI (NSTEMI) or stable angina pectoris (SAP) [27]. Another study showed increased concentrations of sST2 to be an independent predictor of all-cause mortality in patients with stable CAD [16]. Therefore, a large-sample study including patients with SAP and ACS is urgently needed to further demonstrate the predictive value of sST2 in CAD patients during long-term follow-up.

### Prognostic value of sST2 in cardiovascular disease

Previous studies have suggested that sST2 might be a potential biological marker for mechanical overload in the heart. sST2 was shown to be markedly upregulated in mechanically stimulated cardiomyocytes. Furthermore, sST2 has been demonstrated to predict the outcomes in patients with HF [12, 14, 17, 18]. Recent evidence has suggested that sST2 may be predictive in patients with ACS [28, 29]. According to research by Eggers KM, sST2 levels were elevated early in NSTE-ACS and predicted 1-year mortality [10]. A study by Wang YP showed that the serum levels of sST2, IL-33 and BNP were positively correlated with MACEs in patients with AMI after PCI [30]. However, no studies have investigated the long-term value of sST2 in the prediction of MACEs or all-cause death in a large population of patients with CAD.

### The underlying mechanisms

The inflammatory hypothesis of atherosclerosis suggests that inflammatory cell signaling drives the formation, development, and eventual instability of atherosclerotic plaques [31]. IL-33 was originally reported to be a modulator of inflammation that tips the balance toward CD4 + T helper-cell type 2-mediated immune responses [32]. The effect of IL-33 on the function of foam cells indicated the protective role of IL-33 in atherosclerosis [33]. sST2 acts as a decoy receptor for IL-33, thus blocking its protective effects. It has been reported that ApoE (-/-) mice treated with soluble sST2 developed significantly larger atherosclerotic plaques in the aortic sinus compared with control mice [34]. Researchers have found that sST2 is specifically expressed in arterial endothelial cells and is involved in the progression of atherosclerosis [35]. These results suggested that sST2 could be a marker of plaque burden and a predictor of future cardiovascular events [36]; therefore, the IL-33-ST2 pathway deserves consideration. Although the above data suggest that sST2 plays a role in the prognosis of patients presenting with ACS, whether sST2 contributes to cardiovascular risk prediction in a large population of CAD patients during long-term follow-up remains uncertain.

To evaluate the prognostic value of a biomarker in CVD, researchers must demonstrate the elevated risk of cardiovascular events associated with higher levels of the new biomarker with adjustment for other established risk factors. The results should be presented as hazard ratios or relative risk estimates obtained by a Cox model and a probability value test of significance of the marker in multivariable models [37]. According to our results, the adjusted HRs for MACEs and all-cause death were 1.36 and 2.01, respectively, in the Cox proportional hazards models after incorporating age, sex, and other clinically relevant covariates. Moreover, in previous studies, the follow-up time for the predictive value of sST2 was relatively short. Brown et al. assessed the prognostic value of sST2 for acute MI, ACS, and MACEs over a short-term follow-up of 30 days [38]. Aldous et al. revisited the prognostic value of sST2 in patients with chest pain over a longer follow-up of 18 months [39]. Two other reports were based on data from 3 clinical trials in patients with STEMI and provided data on the prognostic value of plasma sST2 for 30 days after MI for adverse events, while another study reported the prognostic performance over an average follow-up of 20 months [29, 40, 41]. Our results demonstrated that, after a median follow-up of 6.4 years, a higher level of sST2 was significantly associated with all-cause death and MACEs and provided incremental prognostic value beyond traditional risk factors.

### Prognostic value of sST2 in CAD patients with and without diabetes

In our study, sST2 remained a significant predictor of MACEs in patients both with and without T2DM after adjusting for age, sex and other confounders. The level of sST2 was significantly higher in the diabetes group than the non-diabetes group, consistent with previous findings [19]. For MACEs, the AUC increased from 0.675 to 0.737 among patients with T2DM; the AUC increased from 0.581 to 0.620 in patients without T2DM. For all-cause death, among patients with T2DM, the AUC increased from 0.896 to 0.923. The AUC increased from 0.744 to 0.789. The AUC of CAD patients with T2DM was significantly higher than for those without T2DM, indicating that sST2 had greater predictive value for MACEs and all-cause death in the prognosis of CAD patients with T2DM and provided new evidence for the role of sST2.

Based on Lin’s research, sST2 levels were significantly elevated in patients with diabetes compared with normal control subjects, and each SD log sST2 was associated with a 1.57-fold increased risk of atherosclerosis [19]. Another study showed that sST2 was regulated by the p75 neurotrophin receptor and predicted mortality in patients with diabetes [20]. Durga’s research suggested that elevated levels of sST2 were able to predict mortality and MACEs in ACS patients and an increased risk of MACEs and mortality in ACS patients with diabetes [42]. Hasan’s research indicated that circulating sST2 could be used to establish a cutoff value for cardiometabolic risk/disease in individuals with glycemia in the normal/prediabetes range [43]. In contrast, subclinical cardiac dysfunction was reported to be associated with older age, male sex, and metabolic factors but not with the sST2 level [44]. IL-33 serves as an important local link between tissue injury or metabolic disturbances and a physiological response to limiting and repairing tissue damage [45]. It has been shown that circulating sST2 is associated with markers of liver function and lipid metabolism in severely obese patients and that a reduction in sST2 occurs after successful bariatric surgery, most prominently in patients with diabetes [46]. IL-33 and sST2 are abundantly expressed in adipose tissues, and IL-33 levels are correlated with high BMI, suggesting an association of IL-33 with obesity and diabetes [47, 48]. Based on the above studies, we believe that sST2 might affect metabolism and further affect the prognosis of patients with diabetes, but the underlying mechanism has not been confirmed. ST2L, sST2, and IL-33 are expressed in many tissues, including adipose tissue, and they are increased in obesity. In mouse obesity models, IL-33 plays a protective role by reducing obesity and increasing glucose and insulin tolerance. The IL-33/sST2 axis appears to play a role in metabolic disorders, but this theory is based primarily on in vitro studies and in vivo studies in animals [49, 50]. In vitro, IL-33 reduces lipid storage and the expression of genes involved in lipid metabolism and fat formation. sST2 − / − mice fed a high-fat diet gained more weight and were impaired in insulin secretion and glucose regulation [51]. The functional role of IL-33 has thus expanded from infection to inflammation and metabolic disease, which might also partly explain why sST2 remained a significant predictor of MACEs in patients both with and without T2DM. This exciting area of research deserves further study and has considerable therapeutic implications.

### Limitations

Our study still has several important limitations. First, while the study provided a large, well-characterized study sample with adjudicated outcomes, the research was limited to a single center, and these data represent the results of an observational analysis. As in any observational study, we cannot exclude residual confounding. Second, other promising new biomarkers, such as high-sensitivity troponin I and high-sensitivity troponin T, interleukin 6 and hs-CRP, were not included in our study. The combination of these biomarkers could have greater prognostic value in patients with established CAD. Third, according to our research, higher values of sST2 confer a markedly adverse prognosis, and the underlying mechanisms should be studied. Fourth, the results from the present study, which included only Chinese patients, cannot be extrapolated to other ethnic groups, and further studies are required.

## Conclusions

Higher values of sST2 confer a markedly adverse prognosis characterized by excessive risk of MACEs and all-cause death over a long follow-up period. Our study demonstrated that sST2 is a useful predictor of adverse clinical outcomes in patients with CAD, suggesting that elevation of sST2 might provide long-term prognostic information for CAD patients. In subgroup analysis depending on diabetes status, the diabetes group still had a significantly higher level of sST2, and it remained a significant predictor of MACEs and all-cause death in patients with and without T2DM after adjusting for age, sex and other confounders. The AUC of CAD patients with diabetes mellitus was significantly higher than in those without diabetes mellitus. In summary, measurement of sST2 should be considered part of the approach to risk stratification in CAD patients with and without diabetes during long-term follow-up. sST2 has a high predictive value for cardiovascular adverse events in CAD patients with diabetes, and these findings provide new evidence for the role of sST2.

## Data Availability

The datasets used and/or analyzed during the current study are available from the corresponding author on reasonable request.

## Abbreviations

ACS: Acute coronary syndrome
AUC: Area under the curve
CABG: Coronary artery bypass grafting
CAD: Coronary artery disease
CAG: Coronary angiography
CVD: Cardiovascular disease
DM: Diabetes mellitus
HF: Heart failure
HDL-C: High-density lipoprotein cholesterol
HR: Hazard ratio
IDI: Integrated discrimination improvement
IL-1: Interleukin-1
IL-33: Interleukin-33
IHD: Ischemic heart disease
LDL-C: Low-density lipoprotein cholesterol
MACEs: Major adverse cardiovascular events
MI: Myocardial infarction
NRI: Net reclassification index
NEST-ACS: Non-ST segment elevation acute coronary syndrome
NSTEMI: Non-ST elevation MI
PCI: Percutaneous coronary intervention
ROC: Receiver operating characteristic
SD: Standard deviation
STEMI: ST elevation MI
sST2: Soluble suppression of tumorigenesis-2
TC: Total cholesterol
TG: Triglyceride

## References

[1] Timmis A, Townsend N, Gale C, Grobbee R, Maniadakis N, Flather M (2018). European Society of Cardiology: Cardiovascular Disease Statistics 2017. Eur Heart J.

[2] Pichery M, Mirey E, Mercier P, Lefrancais E, Dujardin A, Ortega N (2012). Endogenous IL-33 is highly expressed in mouse epithelial barrier tissues, lymphoid organs, brain, embryos, and inflamed tissues: in situ analysis using a novel Il-33-LacZ gene trap reporter strain. J Immunol.

[3] Cayrol C, Girard J-P (2014). IL-33: an alarmin cytokine with crucial roles in innate immunity, inflammation and allergy. Curr Opin Immunol.

[4] Molofsky AB, Savage AK, Locksley RM (2015). Interleukin-33 in tissue homeostasis, injury, and inflammation. Immunity.

[5] Schmitz J, Owyang A, Oldham E, Song Y, Murphy E, McClanahan TK (2005). IL-33, an interleukin-1-like cytokine that signals via the IL-1 receptor-related protein ST2 and induces T helper type 2-associated cytokines. Immunity.

[6] Dinarello CA (2005). An IL-1 family member requires caspase-1 processing and signals through the ST2 receptor. Immunity.

[7] Pascual-Figal DA, Januzzi JL (2015). The biology of ST2: the international ST2 consensus panel. Am J Cardiol.

[8] Kolodin D, van Panhuys N, Li C, Magnuson AM, Cipolletta D, Miller CM (2015). Antigen- and cytokine-driven accumulation of regulatory T cells in visceral adipose tissue of lean mice. Cell Metab.

[9] Odegaard JI, Lee MW, Sogawa Y, Bertholet AM, Locksley RM, Weinberg DE (2016). Perinatal licensing of thermogenesis by IL-33 and ST2. Cell.

[10] Eggers KM, Armstrong PW, Califf RM, Simoons ML, Venge P, Wallentin L (2010). ST2 and mortality in non-ST-segment elevation acute coronary syndrome. Am Heart J.

[11] Richards AM, Di Somma S, Mueller T (2015). ST2 in stable and unstable ischemic heart diseases. Am J Cardiol.

[12] Emdin M, Aimo A, Vergaro G, Bayes-Genis A, Lupón J, Latini R (2018). sST2 predicts outcome in chronic heart failure beyond NT-proBNP and high-sensitivity troponin T. J Am Coll Cardiol.

[13] Hughes MF, Appelbaum S, Havulinna AS, Jagodzinski A, Zeller T, Kee F (2014). ST2 may not be a useful predictor for incident cardiovascular events, heart failure and mortality. Heart.

[14] Aleksova A, Paldino A, Beltrami AP, Padoan L, Iacoviello M, Sinagra G (2019). Cardiac biomarkers in the emergency department: the role of soluble ST2 (sST2) in acute heart failure and acute coronary syndrome-there is meat on the bone. J Clin Med..

[15] Pfetsch V, Sanin V, Jaensch A, Dallmeier D, Mons U, Brenner H (2017). Increased plasma concentrations of soluble ST2 independently predict mortality but not cardiovascular events in stable coronary heart disease patients: 13-year follow-up of the KAROLA study. Cardiovasc Drugs Ther.

[16] Dieplinger B, Egger M, Haltmayer M, Kleber ME, Scharnagl H, Silbernagel G (2014). Increased soluble ST2 predicts long-term mortality in patients with stable coronary artery disease: results from the Ludwigshafen risk and cardiovascular health study. Clin Chem.

[17] Aimo A, Januzzi JL, Vergaro G, Richards AM, Lam CSP, Latini R (2020). Circulating levels and prognostic value of soluble ST2 in heart failure are less influenced by age than N-terminal pro-B-type natriuretic peptide and high-sensitivity troponin T. Eur J Heart Fail..

[18] van Vark LC, Lesman-Leegte I, Baart SJ, Postmus D, Pinto YM, Orsel JG (2017). Prognostic value of serial ST2 measurements in patients with acute heart failure. J Am Coll Cardiol.

[19] Lin Y-H, Zhang R-C, Hou L-B, Wang K-J, Ye Z-N, Huang T (2016). Distribution and clinical association of plasma soluble ST2 during the development of type 2 diabetes. Diabetes Res Clin Pract.

[20] Caporali A, Meloni M, Miller AM, Vierlinger K, Cardinali A, Spinetti G (2012). Soluble ST2 is regulated by p75 neurotrophin receptor and predicts mortality in diabetic patients with critical limb ischemia. Arterioscler Thromb Vasc Biol.

[21] Li M, Duan L, Cai Y-L, Li H-Y, Hao B-C, Chen J-Q (2020). Growth differentiation factor-15 is associated with cardiovascular outcomes in patients with coronary artery disease. Cardiovasc Diabetol.

[22] Zhao Q, Zhang T-Y, Cheng Y-J, Ma Y, Xu Y-K, Yang J-Q (2020). Impacts of triglyceride-glucose index on prognosis of patients with type 2 diabetes mellitus and non-ST-segment elevation acute coronary syndrome: results from an observational cohort study in China. Cardiovasc Diabetol.

[23] Wong Y-K, Cheung CYY, Tang CS, Hai JSH, Lee C-H, Lau K-K (2019). High-sensitivity troponin I and B-type natriuretic peptide biomarkers for prediction of cardiovascular events in patients with coronary artery disease with and without diabetes mellitus. Cardiovasc Diabetol.

[24] Cediel G, Rueda F, Oxvig C, Oliveras T, Labata C, de Diego O (2018). Prognostic value of the Stanniocalcin-2/PAPP-A/IGFBP-4 axis in ST-segment elevation myocardial infarction. Cardiovasc Diabetol.

[25] Lyngbakken MN, Myhre PL, Røsjø H, Omland T (2019). Novel biomarkers of cardiovascular disease: applications in clinical practice. Crit Rev Clin Lab Sci.

[26] Libby P, Theroux P (2005). Pathophysiology of coronary artery disease. Circulation.

[27] Demyanets S, Speidl WS, Tentzeris I, Jarai R, Katsaros KM, Farhan S (2014). Soluble ST2 and interleukin-33 levels in coronary artery disease: relation to disease activity and adverse outcome. PLoS ONE.

[28] Salvagno GL, Pavan C (2016). Prognostic biomarkers in acute coronary syndrome. Ann Transl Med.

[29] Shimpo M, Morrow DA, Weinberg EO, Sabatine MS, Murphy SA, Antman EM (2004). Serum levels of the interleukin-1 receptor family member ST2 predict mortality and clinical outcome in acute myocardial infarction. Circulation.

[30] Wang Y-P, Wang J-H, Wang X-L, Liu J-Y, Jiang F-Y, Huang X-L (2017). Roles of ST2, IL-33 and BNP in predicting major adverse cardiovascular events in acute myocardial infarction after percutaneous coronary intervention. J Cell Mol Med.

[31] Zhao TX, Mallat Z (2019). Targeting the immune system in atherosclerosis: JACC State-of-the-Art Review. J Am Coll Cardiol.

[32] Kakkar R, Lee RT (2008). The IL-33/ST2 pathway: therapeutic target and novel biomarker. Nat Rev Drug Discov.

[33] McLaren JE, Michael DR, Salter RC, Ashlin TG, Calder CJ, Miller AM (2010). IL-33 reduces macrophage foam cell formation. J Immunol.

[34] Junttila MJ, Hookana E, Kaikkonen KS, Kortelainen M-L, Myerburg RJ, Huikuri HV (2016). Temporal trends in the clinical and pathological characteristics of victims of sudden cardiac death in the absence of previously identified heart disease. Circ Arrhythm Electrophysiol..

[35] Abulizi P, Loganathan N, Zhao D, Mele T, Zhang Y, Zwiep T (2017). Growth differentiation factor-15 deficiency augments inflammatory response and exacerbates septic heart and renal injury induced by lipopolysaccharide. Sci Rep.

[36] Aimo A, Migliorini P, Vergaro G, Franzini M, Passino C, Maisel A (2018). The IL-33/ST2 pathway, inflammation and atherosclerosis: trigger and target?. Int J Cardiol.

[37] Vasan RS (2006). Biomarkers of cardiovascular disease: molecular basis and practical considerations. Circulation.

[38] Brown AM, Wu AHB, Clopton P, Robey JL, Hollander JE (2007). ST2 in emergency department chest pain patients with potential acute coronary syndromes. Ann Emerg Med..

[39] Aldous SJ, Richards AM, Troughton R, Than M (2012). ST2 has diagnostic and prognostic utility for all-cause mortality and heart failure in patients presenting to the emergency department with chest pain. J Card Fail.

[40] Sabatine MS, Morrow DA, Higgins LJ, MacGillivray C, Guo W, Bode C (2008). Complementary roles for biomarkers of biomechanical strain ST2 and N-terminal prohormone B-type natriuretic peptide in patients with ST-elevation myocardial infarction. Circulation.

[41] Dhillon OS, Narayan HK, Khan SQ, Kelly D, Quinn PA, Squire IB (2013). Pre-discharge risk stratification in unselected STEMI: is there a role for ST2 or its natural ligand IL-33 when compared with contemporary risk markers?. Int J Cardiol.

[42] Jha D, Goenka L, Ramamoorthy T, Sharma M, Dhandapani VE, George M (2018). Prognostic role of soluble ST2 in acute coronary syndrome with diabetes. Eur J Clin Invest.

[43] Hasan A, Aldhahi W (2020). Soluble suppression of tumorigenicity 2 is directly correlated with glycated hemoglobin in individuals with an average glycemia in the normal/prediabetes range. Diabetes Metab Syndr Obes.

[44] Nah E-H, Cho S, Kim S, Cho H-I (2020). Reference interval and the role of soluble suppression of tumorigenicity 2 (sST2) in subclinical cardiac dysfunction at health checkups. J Clin Lab Anal..

[45] Altara R, Ghali R, Mallat Z, Cataliotti A, Booz GW, Zouein FA (2018). Conflicting vascular and metabolic impact of the IL-33/sST2 axis. Cardiovasc Res.

[46] Demyanets S, Kaun C, Kaider A, Speidl W, Prager M, Oravec S (2020). The pro-inflammatory marker soluble suppression of tumorigenicity-2 (ST2) is reduced especially in diabetic morbidly obese patients undergoing bariatric surgery. Cardiovasc Diabetol.

[47] Molofsky AB, Van Gool F, Liang H-E, Van Dyken SJ, Nussbaum JC, Lee J (2015). Interleukin-33 and interferon-γ counter-regulate group 2 innate lymphoid cell activation during immune perturbation. Immunity.

[48] Vasanthakumar A, Moro K, Xin A, Liao Y, Gloury R, Kawamoto S (2015). The transcriptional regulators IRF4, BATF and IL-33 orchestrate development and maintenance of adipose tissue-resident regulatory T cells. Nat Immunol.

[49] Miller AM, Asquith DL, Hueber AJ, Anderson LA, Holmes WM, McKenzie AN (2010). Interleukin-33 induces protective effects in adipose tissue inflammation during obesity in mice. Circ Res.

[50] Molofsky AB, Nussbaum JC, Liang H-E, Van Dyken SJ, Cheng LE, Mohapatra A (2013). Innate lymphoid type 2 cells sustain visceral adipose tissue eosinophils and alternatively activated macrophages. J Exp Med.

[51] Liew FY, Girard J-P, Turnquist HR (2016). Interleukin-33 in health and disease. Nat Rev Immunol.

